# Why Females Do Better: The X Chromosomal TLR7 Gene-Dose Effect in COVID-19

**DOI:** 10.3389/fimmu.2021.756262

**Published:** 2021-11-11

**Authors:** Anna E. Spiering, Teun J. de Vries

**Affiliations:** ^1^ Amsterdam University College, University of Amsterdam and Vrije Universiteit Amsterdam, Amsterdam, Netherlands; ^2^ Department of Periodontology, Academic Centre for Dentistry Amsterdam, University of Amsterdam and Vrije Universiteit Amsterdam, Amsterdam, Netherlands

**Keywords:** TLR7, interferon, COVID-19, X chromosome inactivation escape, gene-dose effect, sex differences

## Abstract

A male sex bias has emerged in the COVID-19 pandemic, fitting to the sex-biased pattern in other viral infections. Males are 2.84 times more often admitted to the ICU and mortality is 1.39 times higher as a result of COVID-19. Various factors play a role in this, and novel studies suggest that the gene-dose of Toll-Like Receptor (TLR) 7 could contribute to the sex-skewed severity. TLR7 is one of the crucial pattern recognition receptors for SARS-CoV-2 ssRNA and the gene-dose effect is caused by X chromosome inactivation (XCI) escape. Female immune cells with TLR7 XCI escape have biallelic TLR7 expression and produce more type 1 interferon (IFN) upon TLR7 stimulation. In COVID-19, TLR7 in plasmacytoid dendritic cells is one of the pattern recognition receptors responsible for IFN production and a delayed IFN response has been associated with immunopathogenesis and mortality. Here, we provide a hypothesis that females may be protected to some extend against severe COVID-19, due to the biallelic TLR7 expression, allowing them to mount a stronger and more protective IFN response early after infection. Studies exploring COVID-19 treatment via the TLR7-mediated IFN pathway should consider this sex difference. Various factors such as age, sex hormones and escape modulation remain to be investigated concerning the TLR7 gene-dose effect.

## Introduction

Early in the pandemic, male sex was identified as a risk factor for hospitalization and mortality after SARS-CoV-2 infection (1). While behavioral and socio-economic factors are implied, immunological differences between the sexes may be more important in this disparity. Females typically show a stronger immune response than males and possible factors that could explain this phenomenon are X chromosomal genes, immunomodulatory functions of sex hormones and sex-dependent expression of susceptibility genes (2, 3). Females have better clinical outcomes than males following sepsis and infection (4–7). For instance, males suffer from more severe and intense disease after hepatitis B virus (HBV) or Epstein Barr virus (EBV) infection (7). The immune response differs between males and females in various ways, ranging from innate recognition to downstream adaptive immunity, resulting in distinctly different cytokine responses to infection. Induction of pattern recognition receptors (PRRs), subsequent interferon (IFN) and antibody production is higher in females than males. Sex hormones display immunomodulatory activity at various levels, and immune response to viruses can vary with natural hormonal fluctuations in females. Novel research suggests also an important role for sex chromosomal gene that can modulate differences in the immune response or cause differential expression of disease susceptibility genes such as class I and II MHC glycoproteins (3).

On the other hand, autoimmune diseases are predominantly present in females (4–6, 8). For instance, the incidence of systemic lupus erythematosus (SLE) is almost nine-fold higher in females and male Klinefelter patients (47, XXY), but rarely develops in female Turner patients (45, X0) (9), indicating the importance of the X chromosome in SLE and immunity. The X chromosome is home to many genes directly or indirectly related to immunity and variability in expression of X chromosomal genes is often associated with sex-related immune disparities

The sex-bias observed in the COVID-19 pandemic fits this pattern. An X chromosomal gene of interest is TLR7, identified as a pattern recognition receptor (PRR), recognizing ssRNA viruses such as SARS-CoV-2. It is one of the PRRs involved in type 1 interferon (IFN) production in COVID-19 (10, 11). In the present article, we hypothesize that contribution of beneficial gene expression of the second female X chromosome could partially explain the sex-skewed ICU admission. In particular, this hypothesis and theory article focuses on TLR7 expression and activation and the consequences for males and females in COVID-19.

## Subsections

### COVID-19 and TLR7

Severe acute respiratory syndrome coronavirus 2 (SARS-CoV-2) is the single-stranded RNA (ssRNA) virus that causes COVID-19. The spike protein on the viral envelope binds to the angiotensin-converting enzyme receptor 2 (ACE2) on the host cell. ACE2 has a systemic function in lowering blood pressure and is expressed in most organs, but abundantly in lung respiratory epithelium. The spike protein is activated by the serine protease 2 transmembrane protein (TMPRSS2) on the host cell, allowing for viral particles’ internalization. The viral RNA is subsequently released into the host cytoplasm, from where it can move to the host ribosome for production of new virions to infect other cells. Alternatively, it can enter the endosome (2, 12). Various cellular sensors can recognize SARS-CoV-2 ssRNA or other intermediates of viral replication and induce downstream signaling to activate the innate immune response. At the level of the lung epithelium, these sensors are cytoplasmic RIG-I and MDA5 (13, 14). The inflammatory mediators produced by the epithelium are picked up and further amplified by PRRs in innate immune cells (15), such as TLR7 and TLR8.

**Figure 1:**
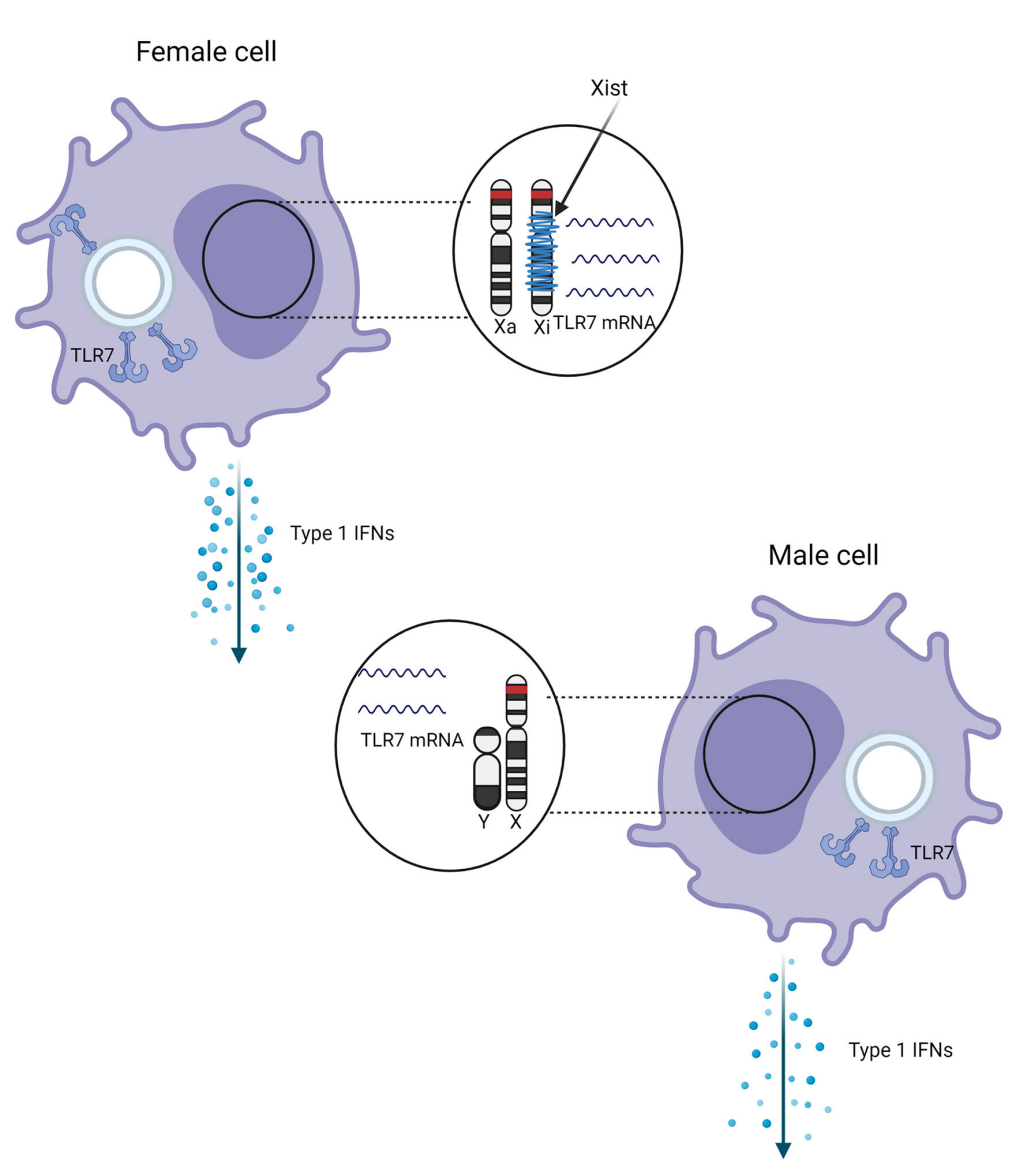
Schematic representation of the effect of sex and X chromosome inactivation escape on the TLR7 expression. TLR7 gene (red) on the inactivated X chromosome (Xi) wrapped with Xist (blue) escapes inactivation. Escape from distal X chromosome inactivation in female pDCs may result in more TLR7 mRNA and protein compared male cells, resulting in higher production of type 1 IFNs.

Toll-Like Receptor 7 (TLR7), and also TLR8, is a PRR encoded on the X chromosome. TLR7 is expressed at the endosomal membrane of plasmacytoid dendritic cells (pDCs) and B cells and to a lesser extent in monocytes and macrophages (16). TLR7 can recognize ssRNA derived from viruses and bacteria in humans, and can also respond to RNA-associated autoantigens (17). Following detection of ssRNA, TLR7 activates the MyD88-dependent pathway, activating both the nuclear factor kappa Beta (NFκB) and the type 1 interferon (IFN-α and IFN-β) pathway in pDCs (18). This process is dependent on the gene product of CXorf21, an X chromosomal gene encoding for the TLR adaptor interacting with endolysosomal SLC15A4 (TASL) protein which stimulates nuclear migration of interferon regulatory factor 5 (IRF5) (19). IFN-α transcription in pDCs is further dependent on the nuclear migration of IRF7 (18), as shown in **Figure 1**. pDCs are the most potent IFN producers and while other cells can also produce IFNs, pDCs function as the primary type 1 IFN producers during viral infections (20). In macrophages, TLR7 stimulation can lead to production of pro-inflammatory cytokines (21).

The most important type 1 IFNs are IFN-α and IFN-β. The production of type 1 IFN is essential for the antiviral response, especially in the early stages of infection. Type 1 IFNs have essential functions: activating an antiviral state in infected cells to limit the viral spread, restraining innate immunity, controlling pro-inflammatory pathways, and activating adaptive immunity. They exhibit both direct and indirect antiviral activity. IFNs bind the interferon-alpha/beta receptor (IFNAR) at infected cells, which initiates the JAK-STAT signaling pathway and the following cascade destroys both host and viral RNA. Viral replication and production are directly restricted by the induction of IFN-stimulated genes (ISGs) (22–24).

TLR7 has been implicated as PRR in SARS-CoV-1 and Middle East Respiratory Syndrome Coronavirus (MERS-CoV) infections (25). These related viruses possess a high number of binding motifs for TLR7 and bioinformatic analysis showed that the SARS-CoV-2 genome has even more ssRNA motifs that can potentially interact with TLR7 than its family member SARS-CoV-1 (23). Early in the COVID-19 pandemic, the importance of TLR7 after SARS-CoV-2 infection was first demonstrated in a clinical setting by van der Made et al. (10). After four young males (two brother pairs from unrelated families) without medical history were admitted to the intensive care unit (ICU), a genetic cause was suspected. In all four patients, different TLR7 deleterious variants and concurrent low levels of type 1 IFN were identified. Loss of function was confirmed when experiments with primary PBMCs showed no upregulation of TLR7 and a failure to induce IFN-inducible genes (ISGs) after stimulation with a TLR7 agonist (10). Later, more such cases of deleterious TLR7 variants in severe male patients have been described (11, 26, 27), suggesting that a loss-of-function of TLR7 could explain some cases of severe COVID-19 which result in functional defects of type 1 IFN. Likewise, single nucleotide polymorphisms (SNPs) could alter TLR7’s effectivity.

As pulmonary epithelial cells do not express TLR7, they alone are not sufficient for the defense against SARS-CoV-2; TLR7 deficiency was pathogenic in patients by impairing the production of large amounts of type 1 IFNs by pDCs (11). After infecting lung epithelial cells, innate phagocytic cells such as pDCs are recruited and can phagocytose the infected cells and produce type 1 IFNs after PRR stimulation. The clinical observations of deleterious TLR7 mutations and a poor type 1 IFN response identify TLR7 as an important PRR in the immune response against COVID-19.

Since TLR7 is located on the X chromosome, mutations in TLR7 will affect males more than females, who bear two X chromosomes per cell. TLR7 deficiency has been reported as a genetic mediator for severe COVID-19 especially in younger males, with percentages of 1-2% found across cohorts (10, 11, 26, 27). Therefore, genetic screening for TLR7 primary immunodeficiency was recommended in young males with severe COVID-19 in the absence of other relevant risk factors (11, 26, 27). Despite the observed prevalence in young males, the penetrance of deleterious TLR7 mutations may be worse in older patients, as both the pDC number and functional IFN secretion decreases with age, which was associated with a reduced number of pDCs expressing TLR7 (28).

### X Chromosome Inactivation

The TLR7 gene is encoded at the distal end of the X chromosome. To avoid double dosage of X chromosomal genes, one female X chromosome is epigenetically silenced in early fetal development. It is random which of the two parental (either maternal or paternal) chromosomes is inactivated, and females are therefore functional mosaics for the active X chromosome (Xa) and the inactive X chromosome (Xi) (29–31). This process is called X chromosome inactivation (XCI). An early study suggests that exactly one chromosome is entirely inactivated in diploid, and half of the X chromosomes in tetraploid cells (32). This view has been modified: certain genes escape from X chromosome inactivation (see later on).

The X inactivation center (Xic) at the centromere controls XCI and codes for several proteins and RNAs that form a nuclear complex during XCI, of which the long non-coding RNA (lncRNA) X inactivation specific transcript (Xist) is a critical member. Xist is expressed only from the inactive X chromosome (Xi) and wraps around the X chromosome, turning it into the inactive Barr body during interphase (31, 33). Recent studies show that XCI is not complete. This phenomenon, XCI escape, could cause extra gene expression of some genes located on the female X chromosome (34, 35) and is a source of (tissue-specific) sex differences (35). The exact mechanism of XCI escape is still unclear. Most genes that escape XCI are at the distal end of the X chromosomes’ short arm, suggesting that the chromosomal location is relevant for escape susceptibility (36). Since the Xist gene is at the centromere, it has been suggested that the direction of Xist RNA wrapping around the X chromosome is from the center outwards (35, 37, 38). The degree of escape is variable between genes, tissues, and individuals, but this variability is poorly characterized (39, 40). Moreover, it is unclear to what extent the X chromosome inactivation state is variable or stable throughout the course of life (41).

Approximately 15 to 20% of human X chromosomal genes escape inactivation and are transcribed from both X chromosomes. Escape is characterized when the Xi alleles’ mRNA or protein expression is at least 10% of that of the Xa allele (42). XCI escape can be studied in various ways, such as bulk RNA-sequencing (RNA-seq) to assess differential allelic expression or RNA fluorescence *in situ* hybridization (RNA FISH) to detect nascent RNA transcripts. XCI status can also be examined indirectly *via* epigenetic markers such as methylation patterns (43).

### Evidence for XCI Escape of TLR7

TLR7 escapes XCI in different female immune cells: pDCs (20, 44), monocytes and B cells (44). A study by Souyris et al. in 2018 was the first to demonstrate the XCI escape of TLR7 in primary human cells. Souyris et al. confirmed biallelic mRNA expression of TLR7 in 30% of pDCs, monocytes, and B cells from healthy females and male Klinefelter Syndrome (KS) patients (47, XXY). TLR7 transcription from both X chromosomes was observed using RNA FISH in B cells, confirming XCI escape. The relative abundance of TLR7 mRNA transcripts in female biallelic B cells was up to 50% higher than male monoallelic B cells. Western blot testing confirmed this at the protein level: a 1.38- and 1.31-fold increase in TLR7 protein was found in biallelic peripheral blood mononuclear cells (PBMCs) compared to female monoallelic PBMCs and male cells, respectively. Additionally, female B cells differentiated more efficiently into plasmablasts than male B cells upon TLR7 stimulation. None of these effects were observed upon TLR9 stimulation.

**Figure 2:**
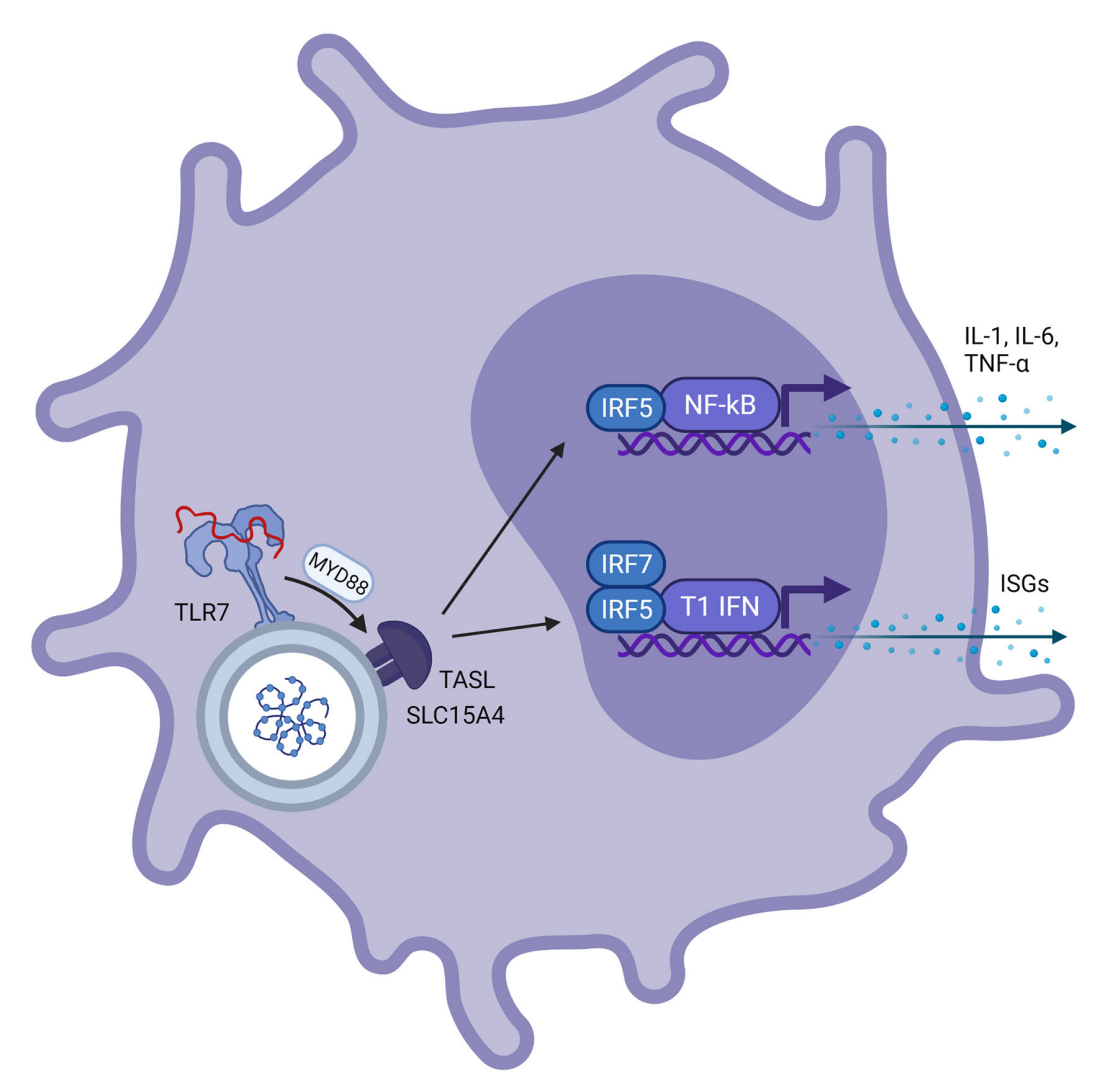
Schematic representation of a plasmacytoid dendritic cell with viral ssRNA in the endosome. This is recognized by TLR7 on the endosomal membrane. *Via* the MYD88 pathway, ‘TLR adaptor interacting with SLC15A4 on the lysosome’ (TASL) is activated which is needed for recruitment of Interferon Regulatory Factor 5 (IRF5), a transcription factor involved in transcription of NF-kβ and type 1 interferons. This results in further transcription of IL-1, IL-6 and TNF-α for NF-kβ and interferon stimulated genes for type 1 interferons, respectively.

Souyris et al. (44) hypothesized that the increased TLR7 dosage would result in increased TLR7-induced type 1 IFN levels, which was confirmed by Hagen et al. (20). This study confirmed 30% female pDCs with biallelic expression and a similar 50% higher abundance of TLR7 mRNA transcripts. Moreover, Hagen et al. observed that biallelic pDCs showed significantly higher type 1 IFN mRNA and protein in the first 2 hours after TLR7 stimulation with a TLR7 agonist (2020). As visualized in **Figure 2**, these results confirmed that human female pDCs produce more type 1 IFN after TLR7 stimulation, showing that TLR7 XCI escape is correlated with the antiviral type 1 IFN response.

Before these two publications, it was already established that female pDCs produced more type 1 IFN after TLR7 stimulation and expressed more interferon alpha/beta receptors (IFNAR) than male pDCs (45–48), especially in females after puberty (49). Similarly, Berghöfer et al. found higher induction of IFN-α in female PBMCs stimulated with TLR7 agonists and not by TLR9 agonists, confirming a skewed and beneficial TLR7 response which could be explained by XCI escape. However, this could not be confirmed in immortalized B-cell lines (46). Others did not investigate XCI escape as a cause of their observations and sought other explanations. Estrogens were suggested as a possible cause of increased IFN signaling in females, as *in vitro* blocking of estrogen receptor (ER) signaling inhibited TLR7-mediated type 1 IFN production by pDCs. However, when female pDCs were transplanted into male mice, an enhanced TLR7-mediated type 1 IFN production was still seen, suggesting that female sex hormones and the number of X chromosomes independently contributed to the enhanced type 1 IFN production (48). Webb et al., who compared genetic deviations with more than one X chromosome, also showed that increased IFN production was dependent on the number of X chromosomes present in the cell (49). A study by Sarmiento et al. found slightly contradictory results: they observed that stimulated female and Klinefelter syndrome (KS) PBMCs had higher TLR7 and IFN-α mRNA levels than male and Turner’s syndrome female PBMCs (45, X0), supporting TLR7 XCI escape, but the KS cells did not show higher IFN-α levels after TLR7 stimulation. In comparison, the female cells showed higher levels compared to the male and TS cells: in the case of the KS cells with two X chromosomes, their higher TLR7 expression by XCI escape did not result in higher IFN, while it did in the female cells with two X chromosomes. Accordingly, the increased IFN production was not dependent on the number of X chromosomes (50), opposite to what others observed (49). One of the explanations provided by Sarmiento et al. is that male KS cells were used, which cannot rule out epigenetic modification of TLR7 by testosterone (50). Altogether, these results strongly suggest that increased type 1 IFN secretion after TLR7 stimulation in female pDCs is linked to biallelic TLR7 expression by XCI escape.

### Possible Implications of TLR7 XCI Escape

This gene-dose effect may play a role in the COVID-19 pandemic, where a significant sex bias has been observed. A meta-analysis of over 3 million global cases between the 1^st^ of January to the 1^st^ of June 2020 revealed that males are 2.84 times more often admitted to the ICU with severe COVID-19 and die 1.39 more often than females, while there is no difference in the proportion of males and females infected with SARS-CoV-2. Socio-economic, lifestyle, and behavioral factors may be partly responsible for this sex bias, but it is equally likely that differences in males’ and females’ immune responses are the driving factor for various reasons. Firstly, the difference was consistently and ubiquitously observed around the world in the selected time period (1). Additionally, another meta-analysis found that while age is another critical risk factor for severe COVID-19, males have a significantly higher risk of death than females at all ages above 30 years (51) and a similar sex bias had been previously observed in the SARS-CoV-1 and MERS-CoV outbreak (52), two ssRNA viruses closely related to SARS-CoV-2. This is consistent with the male sex bias observed in other ssRNA viruses such as hepatitis C and ebola (1, 53). TLR7 gene dosage is a known risk factor and area of interest in SLE, as overexpressing TLR7 in mice leads to SLE-like disease and TLR7 XCI escape has been connected to the high incidence of SLE in females (9).

Altogether, this suggests an actual biological and immunological difference in response to SARS-CoV-2 infection. XCI escape of TLR7 and subsequent increased expression of type 1 IFN could be part of the immunological explanation for the observed sex differences concerning severe COVID-19 susceptibility.

In COVID-19, the immune response is a double-edged sword. Its role after viral infection is to rapidly recognize and eliminate the virus but a late and uncontrolled immune response can lead to immunopathogenesis, such as observed in severe COVID-19 patients (22, 54, 55). The double-edged sword is particularly evident in the distinct clinical phases of severe COVID-19. Patients who later develop severe COVID-19 often show only mild symptoms early on, with low levels of cytokines, leukocytes, and type 1 IFNs. This early phase of severe COVID-19 is characterized by rapid viral replication without detectable pattern recognition receptor (PRR) and IFN triggering, opposite to what is typically seen in pathogenic ssRNA influenza viruses (56). Later, around 7-10 days after symptom onset, these patients rapidly deteriorate. Levels of inflammatory cytokines and chemokines (IL-1, IL-6 and TNF-α) increase to a delayed peak at this stage while lymphocyte levels are low and inflammatory dysregulation develops into acute respiratory distress syndrome (ARDS) (22, 57–59). These high levels of inflammatory cytokines is also called cytokine storm (CS) and is not uncommon in infectious diseases. It can result in systemic inflammation, organ failure, acute lung pathology, and ARDS, as seen in COVID-19 (22, 24, 55, 57).

Mild patients may induce a type 1 IFN-mediated innate immune response shortly after infection, obstructing viral replication at an early stage (60), opposite to what is seen in patients infected with highly pathogenic influenza viruses (56). Moreover, this is in contrast to severe patients who either show a delayed IFN peak or no peak at all. Continuously low as well as delayed peak levels of type 1 IFNs concurrent with a decreased viral load have been observed, suggesting that cause of the symptoms lies, at this stage of the disease, rather at the immune system than at the level of the initial viral infection (22, 57–59). A reduced type 1 IFN response concurrent with a consistent chemokine production has been observed both *in vitro* and in COVID-19 patients (61). A strong and timely, not delayed IFN response is considered protective. But if this response is uncontrolled and late, the increased cytokine and chemokines, many of which are interferon stimulated genes (ISGs), can contribute to development of ARDS. Increases in IL-6 and TNF-α are independent predictors of COVID-19 severity (62). As chemokines are often expressed by an induction of the NF-kB pathway, these results suggest that the NF-kB pathway is induced more than the type 1 IFN pathway (61). Demonstrating the importance of type 1 IFNs are observations of deleterious IRF7 mutations (63) and auto-antibodies against IFNs (64) associating with mortality in COVID-19 patients. Various studies reported an early IFN peak in mild to moderate patients and low IFN levels associated with severe disease and mortality (56–58, 60, 65–68). This profound inflammation is considered one of the leading causes of severity in the later phases of COVID-19 (22, 55, 57–59, 69). A study investigating immune differences in COVID-19 found that increased severity in male patients correlated to high cytokine levels, which was not the case for the females in this cohort (70). With biallelic expression of TLR7 by XCI escape in at least part of their immune cells, females could be expected to mount a more distinct type 1 IFN response early on after SARS-CoV-2 infection, associated with mild disease and possibly preventing them from progressing to severe disease and immunopathology. This is supported by the observed sex bias early in the pandemic of increased hospitalization and mortality in males compared to females and data about the differential role of the immune system in COVID-19. An experimental or clinical study that examines sex differences in the TLR7 and type 1 IFN response in COVID-19 is necessary to further explore this hypothesis.

Additionally, more recent SARS-CoV-2 strains and long-COVID are topics of interest. Analysis of 4000 COVID-19 cases in an app in which individuals can self-report their symptoms showed that female sex is associated with long COVID prevalence (71). This could be explained by the observation in a recent preprint of elevated IFN levels in long COVID patients at 8 months after infection (72), as the IFN response is usually more distinct in females (45–48).

The observation of the sex bias in COVID-19 severity was done in cases up until June 2020 (1), and with a rapidly mutating virus, this may be different in newer strains. While studies into more recent SARS-CoV-2 strains are slowly appearing, it is already emerging that the delta (B.1.617.2) variant come with an increased hospitalization risk (73). Studies about a possible sex bias in this variant have, to the best of our knowledge, not yet been published.

X linked differences in TLR7 expression may not only contribute to COVID-19 susceptibility, but also to hepatitis C virus (HCV) or human immunodeficiency virus (HIV) susceptibility (74). Females are more likely to clear HCV in acute infection than males and develop cirrhosis less often in chronic infection. These differences could in part be explained by increased TLR7 signaling, which is beneficial in HCV (74). SARS-CoV-1 and MERS-CoV showed a male sex bias and similarly to SARS-CoV-2, high IFN levels ‘pre-crisis’ and low IFN levels in the ‘crisis’ phase have been observed in these two viruses (56, 75–78). In contrast to COVID-19, females show faster HIV disease progression to acquired immune deficiency syndrome (AIDS) than males, which could be explained by more persistent inflammation in females contributing to immune impairment. It has been demonstrated that females have a higher TLR7-mediated pDC response to HIV-derived ligands, leading to increased type 1 IFN expression, resulting in increased activation of CD8+ T cells (45).

Finally, pediatric cases are of particular interest. Sex differences based on behavioral or hormonal factors may take place later in life, while genetic mechanisms, amongst which XCI escape, may be evident from birth onwards. XCI takes place in the fetus, but it remains unclear when XCI escape takes place. Patterns of XCI escape have been observed already in trophoblastic cells, but in the embryo proper, where XCI escape is probably still absent (43). Initial XCI research already suggested inactivation state may be dynamic over the course of life (79), and later studies suggest that XCI gene silencing may indeed be variable and gene- and tissue specific (41). An experimental mouse study showed age-associated loss of XCI for the *Atp7a* gene: the inactivated X chromosome (Xi) allele of *Atp7a* was silenced in young adult mice but Xi expression levels could rise up to 5% of the active X chromosome (Xa) in older mice (80). Such studies have not been conducted with human cells *in vitro* nor *in vivo*. It would certainly be interesting to examine possible differences in XCI status in the course of female life, for TLR7 and other genes alike (43, 81).

Generally, pediatric autoimmune diseases are more severe and more prevalent in females (82) and male newborns are more vulnerable to infections and death than their female counterparts (83), similar to the pattern seen in adults. Male sex is a risk factor for severe disease after respiratory syncytial virus (RSV) infection, a common childhood infection (82). In pediatric COVID-19, a wide spectrum of severity is seen. Generally, the COVID-19 prevalence is much lower than in adults and it seems that children are less at risk of a severe clinical outcome, although it is yet unclear why (84–90). One possibility is the higher basal expression of MDA5 and RIG-I in respiratory epithelium than adults, which results in a stronger innate response upon SARS-CoV-2 infection (91). Pediatric studies are underrepresented in COVID-19 research (89). Large scale age-stratified COVID-19 studies often do not further specify below the age of 30 or even 50, much less sex-specific studies (1, 92). Interestingly, a review analyzing 12,306 pediatric COVID-19 cases in the USA found no sex bias with equal male and female propensity (84). Another study analyzed age- and sex-stratified excess deaths in 29 high-income countries that possibly associated with the COVID-19 pandemic in 2020 and found no sex difference in excess deaths in children. The deaths in children were at lower or expected levels in this time period (93). In light of XCI escape, a possible explanation for this discrepancy is that escape of XCI increases over age (41, 80). Large COVID-19 cohort studies with clear age- and sex-stratified groups that include XCI parameters could shed light on this issue in the future.

### Treatment Options

Despite the current world-wide increasing vaccination rate (94), effective treatment options are still necessary for those who are not (yet) vaccinated or for breakthrough cases in the already vaccinated population (95). There is a wide array of possible treatment strategies, some of which include the TLR7-IFN axis. TLR7 agonists have been proposed as treatment (22, 25, 96–99), which may prevent the onset of severe COVID-19 in symptomatic patients and even synergize with active antiviral therapy such as remdesivir. TLR7 agonists could be used to stimulate the innate immune response and induce type 1 IFNs and ISGs early in the disease to prevent progression to more severe phases. TLR7 agonists with several modes of administration are already available for other inflammatory diseases with various degrees of efficacy (100) and would need further examination for use in COVID-19, but, no clinical trial was started. Importantly, administration in later phases of COVID-19 is likely to lead to further immune dysregulation and pathology. As this stage is already characterized by immunopathology, further immune stimulation is not likely to relieve symptoms (25, 98). Successful early interventions which prevent late-stage immunopathology could even help to overcome sex-biased COVID-19 severity.

A possible danger that comes with TLR7 agonists is stimulation of both the pro-inflammatory NFkβ and IFN downstream pathways. Other upstream stimulators of IFNs independent of NFkβ could be considered, e.g. STING agonists which have been explored before in oncology immunotherapy and are recently studied in COVID-19 (101).

Since SARS-CoV-2 showed sensitivity *in vitro* to type 1 IFNs, administration of IFNs may be possible and especially beneficial in patients with a very dysregulated IFN response (60, 102) or patients with deleterious TLR7 or IFN mutations (27, 63, 64). No randomized clinical trials have been performed for type 1 IFNs in SARS-CoV-1, but clinical benefits have been suggested by comparing patients’ clinical outcomes. A retrospective cohort of 446 patients showed that early administration of IFN was associated with reduced hospital mortality while late IFN administrated increased mortality and delayed recovery (103), emphasizing that timing of the administration is crucial, similarly to TLR7 stimulation. Experiments showed that the addition of type 1 IFN to SARS-CoV-2 infected cells resulted in a striking decrease in viral replication, significantly more than in SARS-CoV-1 infected cells (60, 61). The potential window of opportunity for therapeutic IFNs is early after infection, since late administration may exacerbate the inflammatory state of advance disease (61, 104). There are currently several single and combination treatment trials with different administration routes of type 1 IFNs going on (24, 78, 104).

SARS-CoV-2 can obstruct IFN production: a transcriptome profiling study, which was conducted using *in vitro* tissue culture, *ex vivo* infection of primary cells and *in vivo* samples from COVID-19 patients and animals, found that SARS-CoV-2 yields lower levels of type 1 IFN, a lower response of ISGs, and high levels of chemokines, in comparison to other respiratory viruses. These results suggest viral IFN suppression (61). Additionally, host risk factors have been found: IFN-neutralizing autoantibodies or genes suppressing IFN production (63, 64, 67, 104). It has to be explored to what extent SARS-CoV-2 can really interfere with IFNs, and what this means for possible treatment strategies.

Interestingly, treatments with TLR7 antagonists have also been proposed (105). These may ameliorate the cytokine storm (CS) in patients in a later stage of COVID-19. Currently, a phase II clinical trial of a TLR7 antagonist (NCT04448756) is being completed, which investigated the antagonist’s possibilities in modulating the CS and immunopathology in severe COVID-19 patients. Suppression of the inflammatory response in later stage COVID-19 is a strategy which can be pursued in various ways. For instance, dexamethasone showed positive results in an early open-label trial (106).

TLR7 and type 1 IFN agonists as well as antagonists have been proposed, depending on the stage and timing of disease, emphasizing the immune system as a double-edged sword. When considering either of these strategies or other immunomodulatory strategies, the difference in TLR7 dosage between males and females needs to be taken into account.

## Discussion

COVID-19 is a disease where the immune system is a double-edged sword, as visible in the different clinical stages. In severe patients, IFN levels are low early on and symptoms are moderate but later, IFN levels rise, and a cytokine storm subsequently occurs. Males are significantly more likely than females to experience severe disease and TLR7 XCI escape in females may be partly responsible for this. TLR7 has been observed to escape XCI in 30% of female immune cells, resulting in biallelic TLR7 expression and increased IFN production compared to males. Severe COVID-19 disease has been associated with a delayed IFN response and increased TLR7-mediated IFN production in females may prevent females from progressing to severe disease.

This sex-determined gene-dose effect may have important implications for managing the COVID-19 pandemic. While sex-based immunological differences are not new (2), sex as a biological variable is often neglected in clinical and especially pre-clinical research, especially in inflammatory diseases (107, 108). Unfortunately, COVID-19 research is no exception. A recent preprint established that the vast majority of many registered clinical trials for COVID-19 do not mention sex or gender as a recruitment criterium nor bring up sex or gender in the description of the analysis phase (109). Fortunately, two major vaccine trials (Pfizer, Moderna) did include sex-disaggregated primary outcome data. However, further analysis of sex-disaggregated adverse events and secondary outcomes was lacking, which could have set a reporting benchmark for future medical intervention reporting (108). Sex-disaggregated data are not provided by all countries and clinical reports about infection or mortality rate are often not sex-disaggregated, further complicating sex-specific analyses (110, 111). The Global Health 50/50 research initiative and the International Center for Research on Women have started the Sex, Gender and COVID-19 Project which tracks sex-disaggregated data and aims to further attract attention for this issue (111).

In various articles calling for investigation of TLR7 agonist or IFN administration early in COVID-19, sex is also not named as a factor to be taken into account (22, 24, 60, 78, 98, 99). This trend is especially surprising considering the gendered hospitalization and mortality rates of COVID-19 and many have called to reverse this (1, 108, 110, 112, 113). While several articles noted the possible sex differences in immune response to COVID-19, only few clinical and experimental studies have been conducted to investigate this further (70). While it is no exception, it is unfortunate to see this, especially in the light of a possible role for higher expression of TLR7 and IFN in females, probably allowing for sex-specific treatments. In treatment based on modulating TLR7 or administrating IFNs, the sex-determined TLR7 gene-dose effect by XCI escape may be especially important considering the importance of proper timing in the different disease phases. While many questions remain about sex differences in the TLR7-mediated IFN response, it is well-established that these differences exist. Not taking these into account in clinical studies is counter beneficial for exploring these differences. For example, it could be studied whether males need a higher dose of TLR7 agonist or antagonist, or IFN administration than females. Another question is the possible difference in TLR7 XCI status between ICU admitted and non-ICU admitted females.

Thus far, two recent studies have shown proof of XCI escape of TLR7 (20, 44) or examined its effect on IFN expression (20). Replications of these results would further support these findings, in conjunction with previously reported increased IFN response of female immune cells to TLR7 stimulation (45, 47, 48). In these studies, it seems likely that some degree of TLR7 XCI escape may play a role. However, little is known about the stability and duration of XCI and XCI escape: TLR7 is no exception. Insights in the mechanisms of Xist spreading and Xist maintenance may provide more answers into stability, modulation and timeline of XCI escape (50). A recent study demonstrated that Xist is continually required in adult human B cells to regulate several X-linked immune genes including TLR7 (81). Single-cell transcriptome data of female patients with either systemic lupus erythematosus (SLE) or COVID-19 revealed Xist dysregulation and overexpression of TLR7 in PBMCs and atypical B cells. These results suggest that Xist RNA maintenance is crucial for TLR7 silencing, and that Xist regulation may be subject to change in disease states such as COVID-19 or SLE and that Xist maintenance may alter TLR7 expression from the X chromosomes (81). Recently, methods for manipulating Xist have been developed for mouse pre-implantation embryos (114). However, there is still much to be learned about Xist maintenance and TLR7 transcription regulation alike, which could open up possibilities for further investigation of XCI escape and possible manipulation.

Other mechanisms than XCI escape may increase the protein dosage. Both TLR7 and TLR8 expression are under the influence of single nucleotide polymorphisms (SNPs) which can impact expression. For instance, the TLR7 SNP rs179008 is carried by approximately 30% of females of European descent and lowers TLR7 protein dosage in pDCs *via* controlling TLR7 mRNA translation. Affected pDCs from both pre and postmenopausal females showed an impaired TLR7-mediated IFN production, but affected male pDCs did not (115, 116). The TLR8 SNP rs3764880 influences expression of TLR8 isoforms in human monocytes and increases the amount of TLR8 protein (21). Additionally, genomic copy number variation (CNV) can also control TLR7 protein dosage, and this CNV has been associated to SLE incidence (117, 118). Thus, the amount of protein dosage is not only under control of epigenetic mechanisms such as XCI escape, but also by CNV and SNPs.

Other X-chromosomal genes may also be implicated in COVID-19, specifically Toll-Like Receptor 8 (TLR8) and TLR adaptor interacting with SLC15A4 on the lysosome (TASL). TLR8 is in many ways related to TLR7: both are encoded on the X chromosome in close proximity to each other. Both are activated by ssRNA and are complementary to each other in the detection of specific ssRNA motifs. TLR8 is absent in pDCs and B cells, but is expressed in myeloid cells such as monocytes, macrophages and neutrophils, where it can drive IFN-β production dependent on IRF5 (21, 119–121). Compared to TLR7, TLR8 is studied to a lesser extent (119, 122). TLR8 has been suggested to escape XCI in mice but this has not been studied yet in humans (123). In SLE, TLR7 dosage as well as TLR8 dosage are important determinants for the observed sex differences (19, 116, 120).

While SARS-CoV-2 ssRNA can trigger a TLR8-dependent inflammatory response from macrophages *in vitro* (124), it was suggested by others that TLR8 is of lower clinical importance in COVID-19, due to its lack of expression on pDCs and that no deleterious TLR8 variants were found in severe patients (11). TASL interacts with lysosomal SLC15A4 to activate IRF5 after TLR7 activation and is an interferon stimulated gene (ISG) (116). Similar to TLR7, both TLR8 and TASL are involved in the etiology of systemic lupus erythematosus (SLE), a female-biased autoimmune disease (19, 116, 120). TASL has not been associated with COVID-19 directly, but indirectly *via* its adaptor function in the TLR7-mediated IFN pathway. Despite its location on the X-chromosome, XCI escape of TASL has not been studied, so it is unclear if there are sex-based dosage differences of TASL.

This article proposed a hypothesis that XCI escape of TLR7 and the resulting gene-dose effect in females is beneficial in COVID-19. Unfortunately, it will prove difficult to establish a causal link between this TLR7 gene-dose effect and increased hospitalization and mortality in males. It seems important to first establish interindividual variation in biallelic expression between mild and severe COVID-19 female patients. Recently, software has been developed to include X chromosomal associations, such as XCI escape, into GWAS analyses (30), but this cannot associate gene dosage, only genetic variation such as SNPs. However, it could be a way forward, for instance in identifying the genetic make-up of female patients who experience severe COVID-19. Various methods are available to study XCI escape (43, 125). RNA sequencing (RNAseq) and expression data to identify XCI escape relies on good methods to differentiate alleles, usually SNP frequencies to distinguish alleles at multiple loci. Single-cell hybridization methods such as RNA fluorescence *in situ* hybridization (RNA-FISH) used by Souyris et al. (44) do not rely on SNPs and can be used to identify cell types within a tissue. Bulk RNAseq methods could be used for larger-scale investigations into quantitative differences in TLR7 expression between males and females, between males and females of various ages, interindividual variability between females and tissue variability.

Age is another risk factor for severe COVID-19. Ageing differentially affects the male and female immune system, with a male trend towards accelerated immune ageing (1, 126). Ageing might also have an effect on XCI escape, which could be established in bulk RNAseq studies. Especially the role of estrogens may be important here, considering that females after menopause experience a significant drop in estrogen levels. Estrogens are known to modulate the female immune response and protect against infection severity *via* upregulating both the innate and adaptive immune response (2). Estrogens and to a lesser extent progesterone can enhance the TLR7-mediated IFN production by pDCs (45, 127) and blocking estrogen receptor (ER) signaling inhibits this IFN production (48, 127). Treating SARS-CoV-1 infected female mice with ovariectomy or an ER antagonist significantly increased mortality, indicating a protective effect of ER signaling in mice (52). Possibly, this protective effect was mediated by TLR7-mediated IFN signaling, but this was not studied. If ER signaling indeed enhances TLR7 signaling, the menopausal estrogen drop could mean that the protective effect of increased TLR7 signaling in females would be partly lost and the female sex bias in COVID-19 would be diminished.

TLR7 is also expressed in stimulated B cells, where it is involved in B cell receptor activation, antibody production and recognizing foreign nucleic acids (128). TLR7 XCI escape was also demonstrated in B cells (44)and increased B cell stimulation and its effects may also be relevant in COVID-19.

Besides the TLR7 gene-dose effect caused by XCI escape, other biological differences between the sexes may influence the sex bias observed in COVID-19. Other factors are differential ACE2 expression, generally increased T cell activity and immunoglobulin production by B cells in females, other X-encoded immune genes, and activity of sex hormones (1, 51).

Interestingly, ACE2, the entry receptor of SARS-CoV-2, is encoded on the short arm of the X chromosome in sites that are known to escape XCI. Despite this localization, XCI escape of ACE2 has not been experimentally demonstrated in the way that TLR7 has been with RNA fluorescence *in situ* hybridization (RNA FISH). Male-female expression differences could be used as an indirect proxy measurement for XCI status. Data from a genotype-tissue expression study shows a tissue-specific heterogenous expression pattern which is *male*-biased in most tissues (40) while XCI escape would show a female-biased expression pattern. However, there are indications that ACE2 expression does not necessarily correlate to ACE2 enzyme activity due to regulation by sex hormones. Therefore, it is necessary to study at the transcriptional, translational and post-translational level (40, 62).

Unexpectedly, ACE2 was recently identified as an ISG (interferon-inducible gene) in respiratory epithelial cells (129). The antiviral type 1 IFN response may allow for viral entry in neighboring cells by upregulating ACE2 expression as SARS-CoV-2 exploits the ACE2-mediated tissue-protective IFN response to gain cellular entry. This raises questions about IFN treatment, because of the possibility of increased cellular entry by increased ACE2 levels. However, patients receiving angiotensin II receptor blockers and ACE inhibitors, widely used antihypertensives that increase ACE2 expression, showed a lower COVID-19 mortality rate compared to patients not receiving these antihypertensives (130). These results imply that ACE2 is important but not sufficient for viral entry, and that other co-receptors such as serine protease 2 transmembrane protein (TMPRSS2) may also be needed. Of interest, IFN-β increases ACE2 expression, while IFNα does not, suggesting that the type 1 IFN subtypes may have distinct functions (130).

Various other immune genes are encoded on the X chromosome and mutations in X-linked immune genes are known to affect males more than females. For instance, a mutation in interleukin-2 receptor subunit gamma (IL2RG) causes severe combined immunodeficiency (SCID). Males with SCID have impaired cellular and humoral immunity, resulting in increased susceptibility for infections, with a symptom onset before the age of one (131). Likewise, other X-linked immune genes may add to the observed sex bias in COVID-19, possibly by escape of XCI. IL-1 receptor-associated kinase-1 (IRAK-1) is the most studied gene regarding the sex bias in inflammatory responses. It escapes XCI, favoring an increased NFκB-dependent gene transcription response in females (132). CD40L and CXCR3, which have an important role in the T cell response, are both encoded at the X chromosome and escape XCI. It is unknown if the sex-differential role of T cells has a role in COVID-19 (51).

Additionally, The X chromosome encodes for 10% of human microRNAs (miRNAs), many of which are associated with modulation of the immune response. Dysregulated expression of miRNAs has been associated with various inflammatory diseases and may also contribute to the sex differences in COVID-19 (54, 132) but there is yet too little evidence to attribute specific sex-typical immune responses to X-linked miRNAs (2).

To conclude, there are clear indications for an X chromosomal TLR7 gene-dose effect in COVID-19. As sex-determined variables are currently understudied, and the effects of other X-related immune genes and TLR7 modulators are unclear, future research should consider to shed more light on the sex bias in COVID-19.

## Data Availability Statement

The original contributions presented in the study are included in the article/supplementary material, further inquiries can be directed to the corresponding author.

## Author Contributions

Research topic was mutually agreed on by both authors, research was primarily conducted by AS, writing was initiated by AS. AS was supervised by TV during the research period, TV supervised and corrected writing. All authors contributed to the article and approved the submitted version.

## Conflict of Interest

The authors declare that the research was conducted in the absence of any commercial or financial relationships that could be construed as a potential conflict of interest.

## Publisher’s Note

All claims expressed in this article are solely those of the authors and do not necessarily represent those of their affiliated organizations, or those of the publisher, the editors and the reviewers. Any product that may be evaluated in this article, or claim that may be made by its manufacturer, is not guaranteed or endorsed by the publisher.
